# Antimicrobial surfaces: A need for stewardship?

**DOI:** 10.1371/journal.ppat.1008880

**Published:** 2020-10-15

**Authors:** Sam S. Cassidy, David J. Sanders, Jim Wade, Ivan P. Parkin, Claire J. Carmalt, Andrew M. Smith, Elaine Allan

**Affiliations:** 1 Materials Chemistry Research Centre, Department of Chemistry, University College London, London, United Kingdom; 2 Department of Microbial Diseases, UCL Eastman Dental Institute, Royal Free Campus, University College London, London, United Kingdom; 3 Infection Sciences, King’s College Hospital, London, United Kingdom; McGill University, CANADA

## The impact of emergent pathogens

Since the 1970s, more than 1,500 new pathogens have been discovered, and many of these have had major impacts on public health [1]. Healthcare-associated infections (HAIs) account for 37,000 deaths in Europe and 99,000 deaths in the United States of America annually and are associated with a financial loss of €7 billion in the EU and US$ 6.5 billion in the US [2]. Many of these deaths are attributed to multiple drug-resistant (MDR) pathogens, which evolved due to the overuse of antimicrobial drugs. Consequently, MDR pathogens are now considered a global threat to public health [3,4]. Historically, MDR infections have been nosocomial, but since the early 2000s, there has been a rise in community-acquired pathogens such as meticillin-resistant *Staphylococcus aureus* (MRSA), which are now detected in public areas such as public transport, airports, and daycare facilities [5–7].

The recent emergence of Middle East Respiratory Syndrome Coronavirus (MERS-CoV), Zaire ebolavirus, and Zika virus led many experts to believe that a pandemic was inevitable and to warn that we were ill-prepared [1]. Severe Acute Respiratory Syndrome Coronavirus 2 (SARS-CoV-2), the agent responsible for the current Coronavirus Disease 2019 (COVID-19) pandemic, is, together with MERS-CoV, Ebolavirus, and Zika, a zoonotic disease. Although the true social and economic impact of the current pandemic is not yet known, it has clearly had a major effect on day-to-day life around the world and on the global economy. Given humanity’s interference with and encroachment into the natural world, COVID-19 is unlikely to be the last zoonosis or pandemic to challenge the world, and the surge in antimicrobial resistance (AMR), predicted following the increase in invasive procedures and antibiotic prescription during the current pandemic, is another cause for alarm [1–4,8,9].

It is widely accepted that new or repurposed antivirals are needed to treat COVID-19 disease and that a safe and effective vaccine is essential for mitigating disease severity and controlling the spread of infection [10]. However, both these processes are lengthy and complex and can require years of research to ensure quality, safety, and efficacy. In stark contrast, antimicrobial surfaces are already available and offer a nonspecific and therefore broad spectrum intervention targeting all pathogens, irrespective of type, through multiple pathways [11,12]. For these reasons, antimicrobial surfaces may contribute to outbreak control, whilst other interventions, including social distancing measures, pathogen-specific drugs, and vaccines, are established. As most of the world prepares to move out of COVID-19 lockdown, the deployment of antimicrobial surfaces as a precautionary public health measure against future outbreaks is attractive and warrants serious consideration. However, we will argue in this article that indiscriminate use of such surfaces is not without risk and potentially counterproductive. To ensure that antimicrobial surfaces are purely advantageous, we argue that they must undergo a thorough investigation of their potential to induce AMR, that the application of antimicrobial surfaces in the home should be carefully considered, and that a system of ‘stewardship’ is a prerequisite for application in healthcare.

## Pathogen transmission and the nature of antimicrobial surfaces

Whilst direct person-to-person contact is clearly important in pathogen transmission, the spread of pathogens via contaminated surfaces is also significant [13,14]. Touch surfaces may permit the survival and multiplication of bacterial pathogens and the transmission of both bacterial and viral pathogens between hosts [15,16]. Whilst thorough cleaning can help reduce the pathogen load on surfaces, cleaning protocols often fail to completely decontaminate surfaces [17–20]. One study done across 27 intensive care units using a fluorescent tracer found that a basic cleaning protocol resulted in disinfection of only 48.1% of all surfaces, with common touch surfaces, such as room doorknobs (25%) and bathroom light switches (26%), even less clean [18]. Thus, even after cleaning, there remains a risk of transmission particularly for pathogens with a low infective dose. Tables 1 and 2 show survival times of pathogens on various surfaces [15]. It is worth noting here that pathogen detectability does not necessarily equate to infection risk since there is likely to be a gradual decline in pathogen numbers over time on most surfaces, and the numbers ‘detectable’ at these time points may not constitute an infective dose. These data could be significant for some pathogens; however, if we consider that, for example, London is served by 6 international airports, and pre-crisis, handled a total of 485,000 passengers daily [21]. The majority of these passengers will go on to interact, directly or through contaminated surfaces, with some of the capital’s commuters who together complete 10.9 million journeys daily [22]. Whilst reducing the frequency of direct interaction between people is a strategy being employed in the current pandemic, the use of antimicrobial surfaces is also likely to impact transmission rates by reducing these indirect interactions.

**Table 1:** Persistence of bacterial pathogens on dry common inanimate surfaces (e.g., plastics, stainless steel, or flooring).

Bacterial Pathogens	Initial Inoculation	Period of Detectability (Source)	Associated Sickness
*Clostridium difficile* (spores)	Approximately 1 × 10^6^ CFU*	5 months [23]	Bowel infection, diarrhea
*Escherichia coli*	Approximately 1 × 10^5^ CFU*	36 days [24]	Kidney failure, bloody diarrhea, vomiting
*Klebsiella* spp.	Approximately 1 × 10^5^ CFU*	32 days [24]	Pneumonia, septicemia, meningitis
*Saphylococcus aureus*	Approximately 4 × 10^5^ CFU*	>90 days [25]	Pneumonia, septicemia

*CFU, colony-forming units.

**Table 2:** Persistence of viral pathogens on common dry inanimate surfaces (e.g., plastics, stainless steel, or flooring).

Viral Pathogens	Initial Inoculation	Period of Detectability (Source)	Associated Disease
Influenza	Approximately 1 × 10^4^ TCID_50_*	2 days [26]	Influenza
Norovirus	Approximately 2 × 10^5^ TCID_50_*	7 days [27]	Gastroenteritis
Rhinovirus	Approximately 1 × 10^4^ TCID_50_*	4 days [28]	Common cold
SARS-CoV-2	Approximately 10^5^ TCID_50_*	3 days [29]	COVID-19

*TCID_50_, 50% tissue-culture infectious dose.

COVID-19, Coronavirus Disease 2019; SARS-CoV-2, Severe Acute Respiratory Syndrome Coronavirus 2.

**Fig 1:**
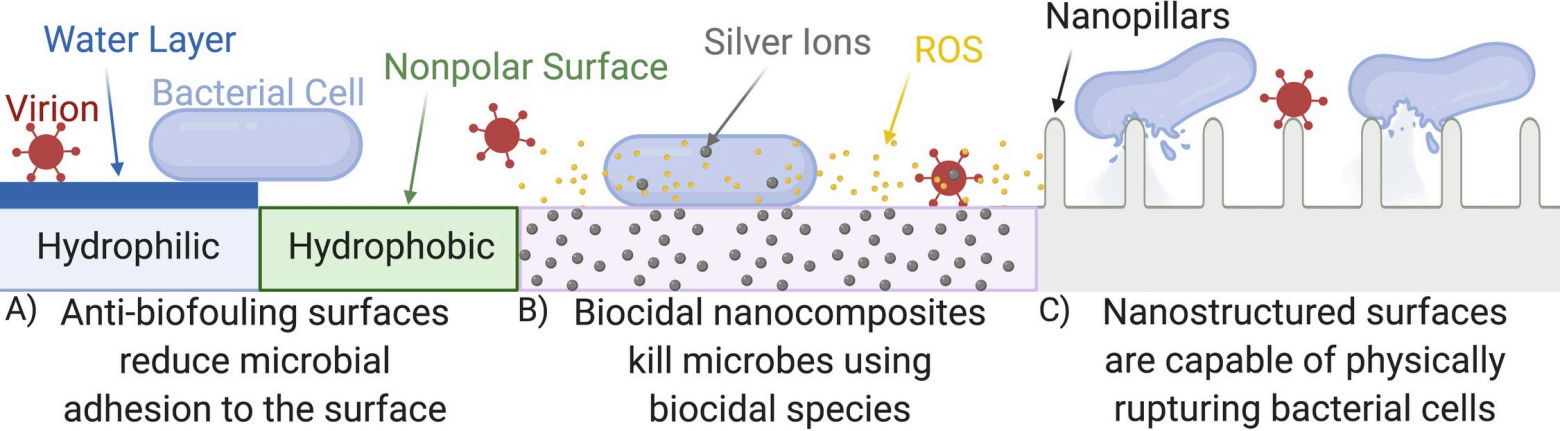
Antimicrobial surfaces operate through a number of mechanisms. (A) Anti-biofouling surfaces use specific surface energies to stop microbial adhesion by deterring adsorption to the surface or by forming a protective layer [36]. (B) Biocidal nanocomposites release incorporated biocidal species or act as a catalyst to produce biocidal species [32,37]. (C) Nanostructured surfaces are capable of physically rupturing bacterial cells; however, further research is required to understand the impact of nanostructured surfaces on virions [35]. ROS, reactive oxygen species.

There are two broadly different, but not mutually exclusive, strategies used in developing antimicrobial surfaces: biocidal surfaces that kill microbes, and anti-biofouling surfaces that reduce microbial adhesion and prevent subsequent biofilm formation (Fig 1). Many commercial antimicrobial surfaces are nanocomposites (bulk materials that incorporate nanomaterials), such as polymers that incorporate silver nanoparticles to confer antimicrobial properties. These nanocomposites are currently being marketed as capable of killing both gram-positive and gram-negative bacteria, as well as inhibiting the H1N1 influenza (swine flu) virus [30,31]. However, silver is an expensive raw material, and the antimicrobial properties of these nanocomposites are largely due to the release of silver ions, meaning that the antimicrobial activity is finite. More recently, nanocomposites are being produced that use the catalytic properties of nanomaterials to drive chemical reactions that produce antimicrobial reagents and are therefore, in theory, self-regenerating [32,33].

In a similar manner to solar cells, semiconducting nanocomposites can use light-driven reactions to produce reactive oxygen species (ROS). However, due to the material band gaps, many antimicrobial semiconducting nanocomposites require UV light or high-intensity white light to reach the excited state needed to produce the ROS required for antimicrobial properties. Recently, a polymer containing crystal violet dye and gold nanoclusters was shown to produce ROS at low flux levels of white light [32]. Through the production of hydrogen peroxide, the surface was bactericidal for both gram-positive and gram-negative bacteria (approximately 5-log reduction in bacterial numbers). Although still under investigation, as hydrogen peroxide is known to be highly effective against both viruses and fungi, this surface is expected to show efficacy against all the major classes of pathogen. Whilst the use of gold and a vivid dye may limit the commercial applications of this product, dye-sensitised nanocomposites offer promise as a persistent antimicrobial surface.

Nanostructured surfaces have also been shown to kill both gram-positive and gram-negative bacteria, including endospores, due to their nanotopography [34,35]. However, the nanotopography of surfaces, such as black silicon, are generally produced using expensive techniques like reactive-ion beam etching which reduces their commercial viability because of increased production cost and time.

With much of the world beginning to exit COVID-19 lockdown, there is an urgent need to test all existing antimicrobial surfaces against SARS-Cov-2 and develop new ones for rapid implementation in appropriate settings. At this juncture, given the importance of the human microbiota for health and the role that environmental microbes play in developing and maintaining that microbiota together with the potential impact of antimicrobial surfaces on AMR, the judicial use of these surfaces is an important consideration.

## The rise of autoimmune disorders and allergies in a world of common infectious disease decline

Whilst there are clearly perceived benefits in using antimicrobial surfaces, questions remain about whether reducing exposure to certain microbes may be detrimental to the development and proper function of the human immune system. Over the last century, the rates of immune-related diseases have risen sharply in developed countries [38]. Interestingly, the trends in developed countries have been mirrored by a simultaneous decline in the prevalence of some infectious diseases, such as hepatitis A, measles, mumps, and tuberculosis. The ‘hygiene hypothesis’, first proposed in 1989, provided a tentative explanation for the shift from infectious diseases to allergies [39]. The idea of being ‘too clean’ was quickly accepted by many and considered to be the underlying cause for the rise in both allergies and immune-mediated diseases. Despite this theory remaining popular with some, subsequent studies have cast a shadow over the original hypothesis.

The discovery and improved understanding of T cell subpopulations, such as regulatory T (T_reg_) cells, in addition to a better knowledge of other immune system components, have led to a greater insight into how the immune system is regulated and how it ‘learns’ to respond appropriately to pathogenic and nonpathogenic microbes and self-antigens [40,41]. Despite reductions in rates of some infectious diseases globally, good hygiene is still regarded as being the first line of defence against recurrent variable pathogens [42,43]. The ‘old friends’ and the ‘disappearing microbes’ hypotheses have superseded the hygiene hypothesis as an explanation for the apparent rise in immune disorders [44,45]. Although providing different perspectives, both theories describe how the human immune system has coevolved with microorganisms, regardless of whether they are commensal, symbiotic, or pathogenic. Loss of these organisms, rather than levels of hygiene, is thought to result in immune system dysfunction, leading to a range of human conditions including immune-mediated diseases, allergy, and even forms of cancer.

## The microbiome, consequences of lifestyle and environment, and links to immune-mediated diseases and host protection

The human microbiome, which comprises all of the bacteria, fungi, viruses, and protozoa that live on and within the human body, plays an important role in human health, starting prior to birth and continuing throughout life. Beginning in utero, humans accumulate microbiota as they interact with the world around them, developing an immune system that takes cues from each encounter [46]. T_reg_ cells suppress the induction of other immune cells, ensuring tolerance to self-, food-, and microbial-antigens, preventing the development of autoimmunity, allergies, and immune-mediated diseases [47]. Lack of exposure to a diverse set of microbes can result in a naïve underdeveloped immune system [44]. Radical changes in hygiene, sanitation, diet, and habitat over the last century are thought to be responsible for impaired immune development. The use of antibiotics, despite their critical role in the fight against pathogenic bacteria, is known to result in long-term disruption to the microbiome [48]. Microbiomes of individuals in developed countries consist of up to 30% fewer species compared to developing countries [49–51]. Consequently, this reduction in microbiota diversity, especially early on in life, has been linked to a rapid rise in allergies and immune-mediated diseases [52]. Studies have demonstrated that both the gut and oral microbiomes of individuals are strongly influenced by cohabitation with genetic ancestry playing no identifiable role [53,54]. The way we live and how we manage our environment through the indiscriminate use of antimicrobial surfaces and reagents could directly impact on our microbiome composition and immune development and inadvertently result in an elevation in the incidence of immune-mediated diseases [52].

**Fig 2:**
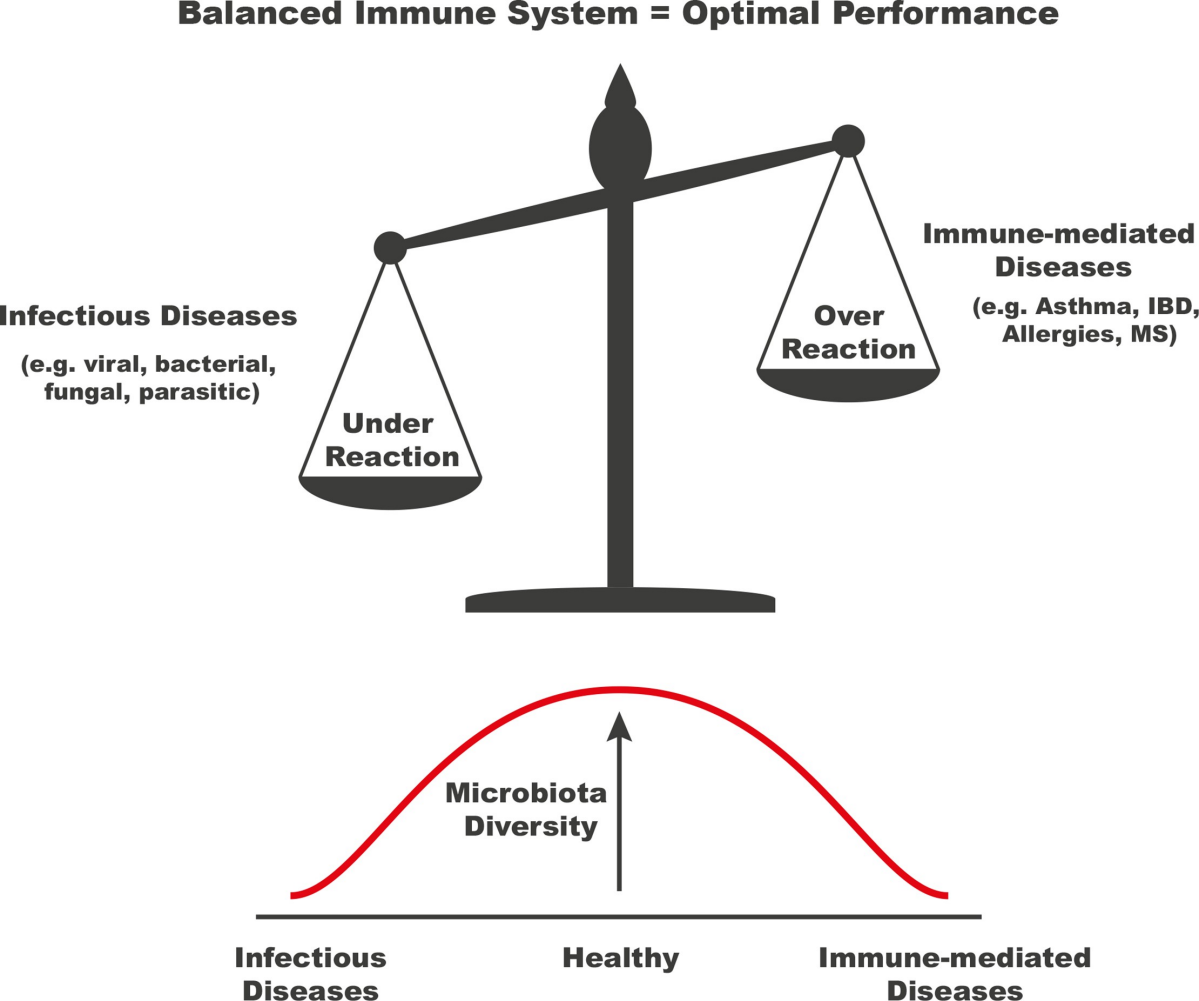
A balanced immune system is one that is both efficient against pathogens and safe to the host. An underactive or weakened immune system increases susceptibility to infectious diseases with the human body unable to fend off pathogenic microorganisms. An overactive or hyperresponsive immune system may react to normally harmless substances in the environment, such as with allergies. Even worse, an overactive immune system may recognise host tissue as foreign and begin to attack the body, such is the case with MS. Whereas exposure to too many pathogens may overwhelm the human body, accumulation and maintenance of a sufficiently diverse microbiota throughout life ensures that the immune system is ‘trained’ to respond appropriately, able to tolerate self- and non-harmful antigens. IBD, inflammatory bowel disease; MS, multiple sclerosis.

The microbiome also has a more direct and active role in host–pathogen interactions, especially viruses. *S*. *aureus* within the respiratory tract has been found to stimulate the immune system and dampen influenza-mediated acute lung injury [55]. Viruses can also exploit the microbiota to promote infectivity. Several naturally occurring gut bacteria have been found to bind multiple versions of norovirus facilitating viral entry [56]. Based on these findings, it could be suggested that the key to a healthy microbiome is not necessarily one with as many microbes as possible but instead a balanced and finely tuned flora beneficial to the host (Fig 2).

## The potential impact on AMR

In addition to a potential impact of antimicrobial surfaces on immune function and microbiome development, another consideration is the risk that selection pressure exerted by antimicrobial surfaces may drive the evolution and spread of AMR. AMR can be intrinsic (for example, in biofilm formed on a surface), or it can be acquired by two principal genetic mechanisms: random mutation or horizontal gene transfer that confer resistance by reducing either the intracellular concentration of the antimicrobial agent, alteration or protection of the molecular target of the antimicrobial or enzymatic inactivation of the antimicrobial agent. Irrespective of mechanism, the end result is that in the sustained presence of an antimicrobial, the resistant subpopulation outcompetes the susceptible population and thus predominates. With regard to antimicrobial surfaces, the risk is not simply that resistance might develop to the specific antimicrobial present in the material but that cross-resistance or co-resistance could occur. Cross-resistance is where one molecular mechanism mediates resistance to more than one antimicrobial—that is, a resistance mechanism induced by the presence of an antimicrobial surface coincidentally imparts resistance to another antimicrobial agent. Co-resistance is a second phenomenon in which the genetic determinants of different resistance mechanisms reside on the same genetic element, thus the selective pressure conferred by the presence of one antimicrobial co-selects for the other. If a determinant for copper resistance, for example, was carried on a plasmid carrying multiple resistance genes specific for different classes of drug, a copper-containing antimicrobial surface has potential to drive the plasmid (and hence multidrug resistance) through a population. A recent article by Pietsch and colleagues [57] reviews studies that indicate a risk of antimicrobial surfaces promoting AMR and considers the evidence in the context of healthcare-related environments.

## Future considerations

Analogous to the impact of the antibiotic era in enabling rapid and significant improvements in human health, the potential of antimicrobial surfaces to help control future outbreaks of infectious disease is enormous. However, just as the antibiotic era has been marred by antibiotic-resistant infections arising from overenthusiastic antibiotic use, it is important that we exert caution in introducing antimicrobial surfaces, and ensure that their use is appropriate. Whilst the benefits of antimicrobial surfaces in high-traffic and high-risk areas, such as transport hubs and healthcare facilities, may seem obvious, it must be borne in mind that exposure to microorganisms from the environment is crucial for microbiome development which, in turn, is needed for proper immune function. Given this knowledge, it would seem appropriate that a system of stewardship is established, at least for clinical application of antimicrobial surfaces, comparable to that recommended for traditional antimicrobials by the National Institute for Health and Care Excellence (NICE) [58]. This system would ensure selection of the most appropriate antimicrobial mechanism, appropriate location, efficacy, durability, stakeholder education, and end-user acceptance. It would also be prudent to consider that introducing antimicrobial surfaces into the healthcare setting may have unpredictable adverse effects. Firstly, regular contact could affect the skin microbiome of the hands of healthcare workers. Products of the normal skin flora—including fatty acids—can be bactericidal and reduce the survival of ‘transient’ opportunist pathogens that may be inadvertently passed between patients on the hands of healthcare workers. Ablation of this skin microbiota may, theoretically, result in persistent colonisation of hands with an abnormal flora. Secondly, surfaces with allergenic potential could result in dermatitis with implications for healthcare workers’ health and practices. Skin affected by dermatitis is prone to colonisation by *S*. *aureus*, an important overt healthcare pathogen. Thirdly, an overreliance on antimicrobial surfaces may induce a false sense of security in the healthcare setting; it is essential that standard hand hygiene practices are maintained. These and other considerations should be addressed in a programme of stewardship.

‘Antimicrobial stewardship’ for traditional antimicrobial agents is now a critical component of modern healthcare and should be the paradigm for policies designed to inform and control the application of antimicrobial surfaces.

Recognising the importance of the human microbiome to health and the need to protect against pathogens whilst enabling exposure to beneficial microbes, the Royal Society for Public Health proposed the concept of ‘targeted hygiene’, whereby cleaning and disinfection is focused exclusively on areas in the home most likely to transmit pathogens, for example, food preparation surfaces such as chopping boards and utensils, sinks, and taps [59]. Integration of antimicrobial surfaces with this type of approach would seem ideal installing them, for example, in food preparation areas and bathrooms but not more generally in floor covering, walls, or furniture.

Manufacturers of antimicrobial surfaces also have a regulatory role to play in ensuring appropriate use. Both concepts of stewardship and targeted hygiene need to be recognised by manufacturers of antimicrobial surfaces to ensure that they are marketed responsibly and installed only where they are likely to impact on infection transmission and not more generally.

In conclusion, the authors call upon industry, national and international policy makers, healthcare professionals, and healthcare agencies (including those responsible for commissioning services and estates management) to recognise that (1) early phase research must address the potential impact of antimicrobial surfaces on AMR before they are widely employed; (2) there is a need for stewardship of antimicrobial surfaces intended for the healthcare setting; and (3) the broader exploitation of antimicrobial surfaces in domestic, industrial, commercial, and transport settings must not be undertaken lightly and requires oversight. With strict regulation and sensible governance, antimicrobial surfaces should become an important and long-lasting addition to the public health armamentarium currently available to control the transmission of infection.
